# Evaluation of small mammal pet supplies offered in German retail under animal welfare aspects

**DOI:** 10.1371/journal.pone.0262658

**Published:** 2022-02-02

**Authors:** Alexandra Bläske, Angela Schwarzer, Magdalena V. Ebner, Hendrikje Gerbig, Sven Reese, Michael Erhard, Anna-Caroline Wöhr

**Affiliations:** 1 Department of Veterinary Sciences, Chair of Animal Welfare, Animal Behaviour, Animal Hygiene and Animal Husbandry, Faculty of Veterinary Medicine, LMU Munich, Munich, Germany; 2 Department of Veterinary Sciences, Chair of Anatomy, Histology and Embryology, Faculty of Veterinary Medicine, LMU Munich, Munich, Germany; University of Life Sciences in Lublin, POLAND

## Abstract

German retailers offer a large variety of accessories for pets. However, not all products are suitable for pet husbandry. Several articles can negatively influence the wellbeing of pets or cause injuries, but empirical studies that evaluate accessories for small pets under animal welfare aspects are rare. In the present study, we assessed articles manufactured or sold in Germany in the product categories pet cages, hay racks, running wheels, exercise balls, harnesses and leashes, tube systems, and hamster bedding. To do so, we searched 28 German websites, visited 50 pet shops and 13 home improvement and garden centers on site and afterwards examined the animal welfare compliance of the products according to various evaluation criteria. Most of the examined products were rated not suitable for pet husbandry and were animal-welfare-adverse. This result applies to 86.1% (*n* = 87) of the 101 assessed running wheel models, 82.7% (*n* = 172) of the 208 assessed pet cage models and 55.6% (*n* = 40) of the 72 assessed hay rack models. The articles in the product categories exercise balls, harnesses and leashes, tube systems, and hamster bedding were also found unsuitable due to animal welfare concerns. Furthermore, we found clear shortcomings regarding article declarations. In some cases, relevant product information (e.g., dimensions) were missing, or the presented information was too general (e.g., rodent cage). Improperly declared pet accessories make it difficult for pet owners to decide whether a product is suitable or unsuitable for the species they keep. A declaration duty for manufacturers of pet products could ensure that German retailers only offer properly declared pet accessories and facilitate the decision for pet owners to purchase products appropriate for the pets they keep. Furthermore, a voluntary product certification for manufacturers would allow retailers to check the animal welfare compliance of articles before including them in their assortment. If a product is unsuitable for pet husbandry because it does not meet the set requirements, it must be considered animal-welfare-adverse and removed from the assortment. As done for the Austrian “animal welfare label,” an independent, qualified third party could do the certification.

## Introduction

According to representative studies, the number of pets in Germany keeps rising steadily. The annually published population study of the “German Pet Trade and Industry Association” (Zentralverband Zoologischer Fachbetriebe Deutschlands e. V. [ZZF]) and the “Industrial Association of Pet Care Producers” (Industrieverband Heimtierbedarf e. V. [IVH]) reported that the number of pets kept in German households rose from 31.6 million in 2016 to 34.0 million in 2019. A closer look at the data for this period reveals, that the number of small mammal pets increased from 5.0 million to 5.2 million whereas the percentage of households keeping small mammal pets decreased from 6% to 5% [1, 2]. These figures indicate a rising trend in Germany to keep multiple small mammals in one household. The increase in keeping small mammals as pets has also been found in other countries [3, 4].

Closely associated with the keeping of pets, especially small pets, are pet supply products for housing, feeding, and exercising the animals because they enable or improve animal husbandry [5]. Pet supply products can for instance be purchased in pet shops, in home improvement and garden centers, or via the internet. With supply articles and accessories (excluding pet food) alone, the German retail sector (grocery and specialist retailers) achieved sales of more than 1 billion euros in 2019. Compared with the previous year’s sales, this is a 2% increase [2, 6]. The sales changes in online retail (supply articles and accessories including pet food) for the same period highlight the enormous and fast growth of the pet industry. Compared with the figures in 2018, the 2019 sales volume in online retail increased by 12.8% and amounted to approx. 705 million euros, as estimated by the ZZF and IVH [2, 6].

Already in the year 2000, Steinigeweg described the assortment of pet supply products as being immense [5]. Furthermore, the increase in market volume for pet products, along with the increasing number of kept pets, is associated with a marked increase in the diversity of offered pet supply articles and accessories [7]. In this context, we must point out that Germany has no legal provisions regarding the design of pet supplies under animal welfare aspects, so that manufacturers can freely design the products [5]. Specific legal provisions regarding a declaration duty are also missing. An exception is feedstuff, which will not be considered in the remaining article. Thus, in the following, the term “pet supplies” includes only accessories, such as pet cages and hay racks. The lacking declaration duty in Germany makes it difficult for pet keepers to distinguish animal-welfare-compliant accessories from products that can possibly or effectively harm the animals. However, according to § 1 of the German Animal Welfare Act [8], animal keepers or caretakers must not use products that can, without acceptable reason, inflict pain, suffering, or injuries on the animals.

Especially in the area of pet keeping, there is a high risk that pet owners do not recognize animal-welfare-adverse pet supplies. [9]. This can be counteracted by so-called "animal welfare labels", which enable pet owners to recognize products that comply with animal welfare standards. For this purpose in Austria, for example, the “Office for Animal-Appropriate Animal Husbandry and Animal Welfare” (Fachstelle für tiergerechte Tierhaltung und Tierschutz) was set up in March 2014, which collaborates closely with the University of Veterinary Medicine, Vienna [10, 11]. The office assesses, inter alia, pet housing systems, accessories, and other items and issues the so-called “animal welfare label” for approved products [12].

The importance of sufficiently large dimensioned pet husbandry systems for the wellbeing and health of animals has repeatedly been highlighted for various small pet species in the relevant scientific literature [13–16]. Dixon et al. found that rabbits in small cages (0.88 m^2^), as compared with rabbits in large cages (1.68 m^2^ or 3.35 m^2^), were less engaged with their environment and spent more time sitting or lying; the authors concluded that undersized cages can jeopardize the wellbeing of the animals [16]. Fischer et al., studying husbandry systems for golden hamsters, reported similar findings; with increasing floor space (largest floor area 1 m^2^), the hamsters showed less wire-gnawing, suggesting increased wellbeing [15]. Despite these empirical findings, deficits considering the housing of small pets in sufficiently large dimensioned cages are found in both private households and pet shops [17–21]. However, the scientific literature lacks precise data on cage models available in retail, especially regarding their declaration and their suitability for housing the declared animal species.

Environmental enrichment allows the animals to better act out their exploration behavior and offers them the opportunity to withdraw from direct observation by the keeper and seek retreat space [13]. An often used enrichment object for small pets is the running wheel [22]. Several authors, especially those concerned with laboratory animal husbandry, studied various animal species and addressed the question which diameter or what type of material is preferred [23–25] or whether the animals use the running wheel at all [26]. The studies showed that mice readily use running wheels not only in a cage [27] but also in nature [26] and that larger running wheels are preferred over smaller ones [24, 25]. However, the results also showed that an inappropriately constructed running surface can lead to injuries for the animals [25, 28, 29]. This is only one example of well-intended enrichment that, due to inappropriate quality, can negatively influence animal health.

Empirical studies that assess and evaluate animal welfare aspects of small-pet supplies available on the market are rare. As part of a research project in Austria in 2007–2008, researchers assessed not only the husbandry conditions of animals in pet shops but also how many shops offered the various assessed pet supply products. The results of the study showed, inter alia, that 69% of the shops sold running wheels with an open axle side and a grid running surface and 50% of the shops offered plastic tube systems [20]. However, detailed descriptions of product properties of the sold accessories and an evaluation considering animal welfare aspects of individual articles were missing.

In Germany, the “Veterinarian Association for Animal Protection” (Tierärztliche Vereinigung für Tierschutz e. V. [TVT]) published a list of animal-welfare-adverse husbandry systems and accessories for birds, small mammals, fish, and reptiles, and this list can serve as basis in the assessment of pet cages and pet accessories [30]. For example, Steinigeweg used this list to exemplarily describe accessory articles available in Germany and assess them under animal welfare aspects [5]. Furthermore, the “German Animal Welfare Association” (Deutscher Tierschutzbund e. V. [DTschB]) wrote a position paper on this topic [31]. However, to date, there exist no detailed data on small mammal pet accessories purchasable in Germany and on their suitability for the animals in regard of animal welfare aspects. Therefore, as part of the EXOPET-II-Study supported by the “Federal Ministry of Food and Agriculture” (Bundesministerium für Ernährung und Landwirtschaft) via the “Federal Office for Agriculture and Food” (Bundesanstalt für Landwirtschaft und Ernährung), we conducted the present study to assess and evaluate under animal welfare aspects the pet cages and accessories purchasable online or on site in pet shops and in home improvement and garden centers. The aim was to assess what percentage of the accessories for small pets offered in the retail are really suitable for pet ownership and what percentage pose a potential threat to the animal welfare and should therefore be considered contrary to animal welfare and unsuitable for pet ownership.

## Materials and methods

The present study is not an animal experiment according to the German Animal Welfare Act (2006). All data were collected in a non-invasive and non-personal way. Therefore, no approval by a competent authority or examination by an ethics committee was needed.

Due to the enormous diversity of pet housing systems and accessories, we decided to restrict the data collection to product groups that are highly likely to contain animal-welfare-adverse articles. For guidance in identifying these product groups, we consulted the “Fact Sheet on Animal-Welfare-Adverse Accessories in Pet Husbandry” (Merkblatt zum tierschutzwidrigen Zubehör in der Heimtierhaltung; Fact Sheet No. 62 [30]) published by the TVT and the “Position Paper on Animal-Welfare-Adverse Accessories and Toys” (Positionspapier: Tierschutzwidriges Zubehör und Spielzeug) written by the DTschB [31]. This selection resulted in seven product categories that were assessed: pet cages, hay racks, running wheels, exercise balls, harnesses and leashes, tube systems, and hamster bedding.

### Assessment of the products sold via online and on-site retail

In the seven product categories, we assessed mostly products available in the German market, but these products are not necessarily produced in Germany. To get an overview of small-pet accessories that are available via online retail, we assessed the pet housing systems and pet accessories offered on 28 German websites from September 2017 to January 2018 and created a product list. This product list contained the product name, sales location, and manufacturer. In addition, product details (dimensions, color, material, construction features) were recorded. Identical articles that were from the same manufacturer and differed only in color were counted only once; identical models of hay racks that differed only in size were also counted only once because size was not evaluated in this product category. When a product was available online and its product information was incomplete or ambiguous, it was ordered so that the missing data for evaluation could directly be assessed at the “Chair of Animal Welfare, Animal Behaviour, Animal Hygiene and Animal Husbandry” (Lehrstuhl für Tierschutz, Verhaltenskunde, Tierhygiene und Tierhaltung) of the Ludwig-Maximilians-University Munich.

The on-site data collection was done in the framework of a study that assessed the state of expertise of sales personnel in shops that sell live animals; throughout Germany, 88 pet shops and 37 home improvement and garden centers were visited from September 2017 to November 2017. With the consent of the store or department manager, photographic documentation of the purchasable pet accessories and cages was recorded in 50 pet shops and 13 home improvement and garden centers. The photographed products were then added to the product list.

### Data editing and data cleansing

For better comparability of the data collected for the herein presented assessment, only those articles available in 2017 were included. At the time of our research, several online shops offered articles that were unavailable. To be able to distinguish if these articles were only temporarily unavailable or if the manufacturer had permanently removed them from the assortment, we asked nine large German manufacturers of pet cages and pet accessories to provide their product catalogues from 2017. Eight of the contacted manufacturers responded to our request, and based on the provided information, products that had the online status “not available” and were no longer manufactured were removed from the product list. Furthermore, articles were removed from the list if they were available only on site in pet shops or home improvement and garden centers and no additional product information could be found (online or in the received manufacturer catalogues) and thus not enough data existed for an evaluation.

### Evaluation of the listed products under animal welfare aspects

Based on the German Animal Welfare Act [8], the “Fact Sheet on Animal-Welfare-Adverse Accessories in Pet Husbandry” from the TVT [30], and the “Position Paper on Animal-Welfare-Adverse Accessories and Toys” from the DTschB [31], evaluation criteria for the seven examined product groups were developed. Because the requirements for the individual products, especially regarding the dimensions of cages and running wheels, can vary according to animal species, the evaluation of animal welfare compliance considered the target animal species for which the product was declared. The following species were considered in the evaluation, as they are among the most commonly kept small mammal pets in Germany: rabbit, guinea pig, ferret/polecat, degu, Mongolian gerbil, chinchilla, golden hamster, fancy mouse, fancy rat and dwarf hamster. Table 1 gives an overview of the product groups and the assessed evaluation criteria. If any of the assessed criterion were not met for a product, it was classified as not suitable for small mammal pet husbandry.

**Table 1:** Product-group-relevant and applied criteria to evaluate animal welfare compliance of accessories offered for small mammal pet husbandry. Basis for the evaluation were the German Animal Welfare Act [8], Fact Sheet No. 62 from the “Veterinary Association for Animal Protection” (Tierärztliche Vereinigung für Tierschutz e. V. [30]), and the position paper from the “German Animal Welfare Association” (Deutscher Tierschutzbund e. V. [31]).

Product category	Evaluation criterion	Criterion not met if:
hay racks	Cover	No cover present
	General design	Injury risk due to protruding screws, instable models, horizontal bars, distance between vertical bars too wide
Harnesses and leashes	General design	Injury risk due to reflex-like flight attempts or constrictions in the chest or belly area due to improperly fitting or incorrectly applied harnesses
Hamster bedding	Digestibility	Not fully digestible
	Material	Made of synthetic material or synthetic fibers
Pet cages	Cage floor area (m^2^) and height (m)	Cage not suitable for housing a hamster or for housing the declared target animal species^1^
	Declaration of animal species	
	Material	Made completely of plastic or glass
Exercise balls	General design	Injury risk due to bumping against a wall or object or falling off of an elevated surface
Running wheels^2^	Running wheel diameter (cm)	Diameter <20 cm for dwarf hamster species
	Declaration of animal species	Diameter <30 cm for all other small pets
	General design	Injury risk due to spaced-apart rungs as running surface or open axle side^3^
Tube systems	Tube diameter (cm)	Tubes too small (diameter)
	General design	Long, poorly ventilated tubes
		Injury risk present

^1^Requirements for the cage floor area for small mammal pets and ferrets are given in Table 2.

^2^Running discs and similar items were not considered.

^3^The side with the wheel suspension.

To evaluate the pet cages, the floor area was calculated based on the available product information (length × width) and the cage height was recorded. If given, both outer and inner dimensions were evaluated. Most of the small mammal species relevant for our assessment study are social species. Thus, the recommended minimum dimensions of the cages were determined assuming that two animals are housed per cage, except for golden hamsters (*Mesocricetus auratus)*, which may be kept singly. As basis for evaluating the minimum cage floor area, we consulted the data from the “Expert Report on Minimum Requirements for Mammal Husbandry” (Gutachten über Mindestanforderungen an die Haltung von Säugetieren [expert report on mammals]) [32], the guidelines in the animal profiles from the “Federal Association for Expertise on Nature, Animal, and Species Protection” (Bundesverband für fachgerechten Natur-, Tier- und Artenschutz e. V. [BNA]) [33–40], and the fact sheets for animal keepers from the TVT [41–47] for each animal species (Table 2). Cage models that were declared for generally housing “rodents” were assigned the minimum dimensions required for housing one golden hamster (0.5 m^2^ floor area, 0.5 m cage height), because these dimensions represent the smallest space allowance required for the study-relevant small mammal species.

**Table 2:** Cage dimension (inner enclosure space allowance) requirements for small mammal pets and ferrets.

Small pet species	Inner enclosure space allowance requirement met if:
Chinchilla^1^	For 2 animals: 2 m^2^ floor area and 1.5 m height
	Per additional animal: +0.5 m^2^ floor area
Degu^1^^,^^2^	For 4 animals: 1.0 m length × 0.5 m width (= 0.5 m^2^ floor area) and 1.0 m height
	Per additional 2 animals: +50% floor area = +0.25 m^2^
Fancy mouse^1^^,^^3^	For 5 animals: 0.8 m length × 0.5 m width (= 0.4 m^2^ floor area) and 0.8 m height
	Per additional animal: +20% floor area = +0.08 m^2^
Fancy rat^1^^,^^2^^,^^3^	For 3 animals: 1.0 m length × 0.5 m width (= 0.5 m^2^ floor area) and 1.0 m height
	Per additional animal: +20% floor area = +0.1 m^2^
Ferret, polecat^2^	For 2 animals: 6 m^2^ floor area and 1.5 m height
	Per additional animal: +1 m^2^ floor area
Golden hamster, dwarf hamster^1^^,^^2^	For 1 animal: 1.0 m length × 0.5 m width (= 0.5 m^2^ floor area) and 0.5 m height
	Per additional animal: +20% floor area = +0.1 m^2^
Guinea pig^1^^,^^2^	For 2 animals: 1.2 m length × 0.6 m width (= 0.72 m^2^ floor area) and 0.5 m height
	Per additional animal: +20% floor area = +0.144 m^2^
Mongolian gerbil^2^	For 2 animals: 1.0 m length × 0.5 m width (= 0.5 m^2^ floor area) and 0.8 m height
	Per additional animal: +25% floor area = +0.125 m^2^
Rabbit^1^	For 2 animals: 1.4 m length × 0.6 m width (= 0.84 m^2^ floor area) and 0.5 m height
	Per additional animal: +20% floor area = +0.168 m^2^

^1^BNA profiles.

^2^TVT fact sheets.

^3^Because no specification was given for additional animals, 20% of the floor area was added for each additional animal (analogous to the specifications for other species [32], personal communication with expert panel).

Tubes systems mostly consist of transparent, straight or bent, and differently long plastic tubes that can be connected via (usually) colored joints with each other, with a burrowing box, or with a cage. For these systems, we evaluated the whole system, not the individual parts. Due to the frequently missing specification of the tube diameter, we often could not differentiate between the different models offered by the same manufacturer. For these manufacturers, we simply recorded whether they offered tube systems but did not assess individual parts or models.

### Data evaluation

The collected data were evaluated by using descriptive statistics assessing the frequency and percentage distribution of the individual categories. We used the software program Microsoft® Excel. The collected data were anonymized before evaluation so that inference on specific internet shops, manufacturers, pet shops, and home improvement and garden centers is not possible.

## Results and discussion

Table 3 gives an overview of the offered products per product group and the numbers of shops and manufacturers offering products in each category.

**Table 3:** Overview of the pet accessories, categorized in seven product groups, offered online in internet shops and on site in pet shops or home improvement and garden centers in Germany. In addition to the number of products, the numbers of shops and manufacturers offering these products are listed.

Sales location	Number of shops	Number of models before data cleansing	Number of models after data cleansing	Number of manufacturers
	Pet cages^1^^,^^2^			
Online shop	28	216	208	20
Pet shop	50	44	36	8
Home improvement and garden center	13	13	13	4
	Hay racks^1^			
Online shop	28	72	72	19
Pet shop	50	27	27	8
Home improvement and garden center	13	10	10	2
	Running wheels^1^			
Online shop	28	101	101	16
Pet shop	50	36	36	11
Home improvement and garden center	13	9	9	3
	Exercise balls^1^			
Online shop	28	25	25	16
Pet shop	3	3	3	2
Home improvement and garden center	n.o.	n.o.	n.o.	n.o.
	Harnesses and leashes^1^			
Online shop	28	50	50	17
Pet shop	21	13	13	3
Home improvement and garden center	4	4	4	1
	Tube systems^3^			
Online shop	28	14	14	13
Pet shop	6	3	3	2
Home improvement and garden center	n.o.	n.o.	n.o.	n.o.
	Hamster bedding^1^			
Online shop	28	10	10	8
Pet shop	22	3	3	3
Home improvement and garden center	4	2	2	2

n.o. = Not offered.

^1^Identical products differing only in color were counted only once per manufacturer.

^2^Cages for housing small pets.

^3^For the tube systems, only the whole systems were assessed per manufacturer, not the individual parts.

### Pet cages

After data cleansing for the following data evaluation, the product category pet cages consisted of 208 cage models that were made by 20 manufacturers and sold via online retail. During the on-site visits, we assessed 36 cage models from eight manufacturers in the pet shops and 13 cage models from four manufacturers in the home improvement and garden centers (Table 3).

**Fig 1:**
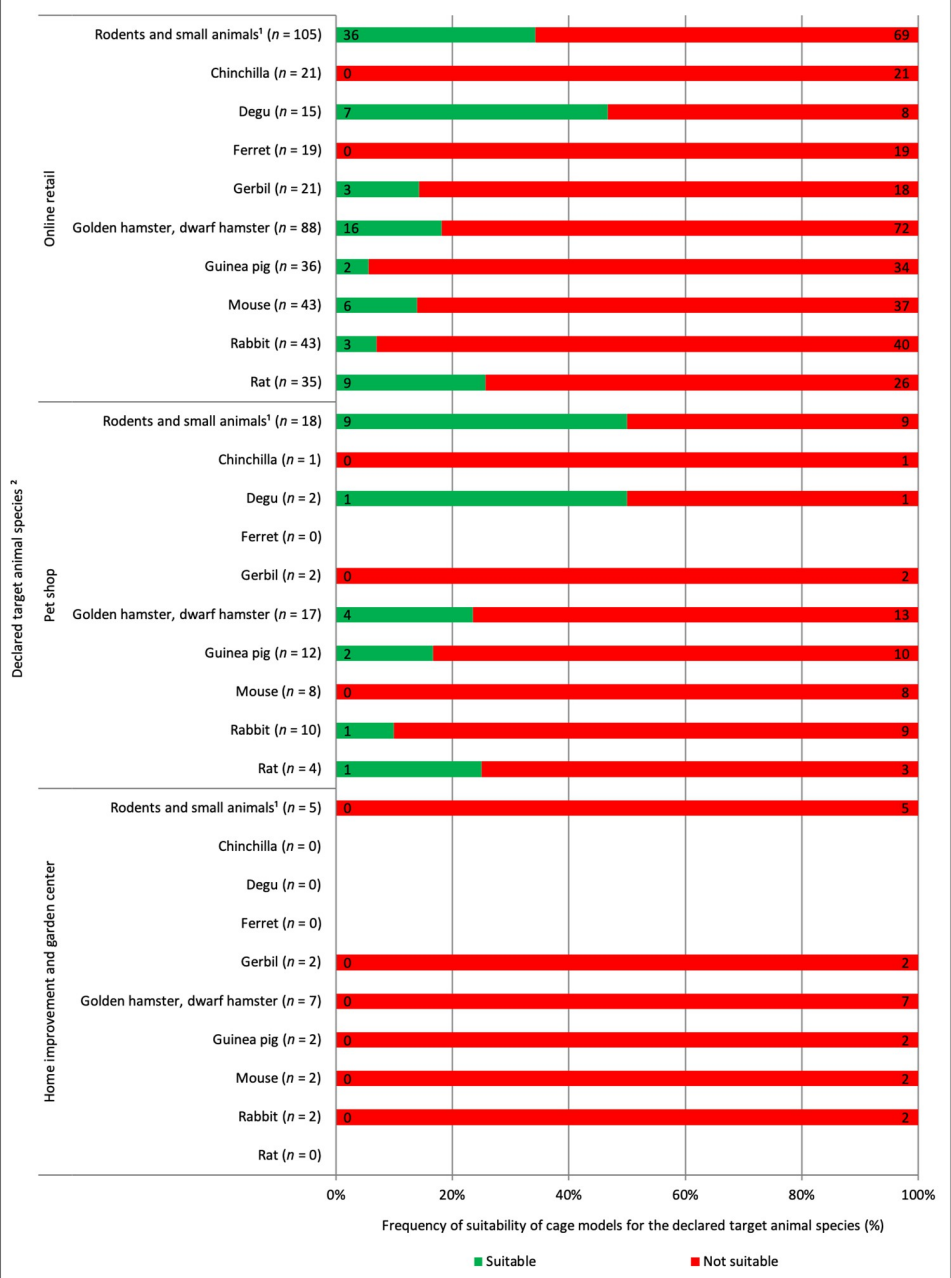
Cage models purchasable in German retail (online via internet shops, on site in pet shops or home improvement and garden centers) and declared for a target animal species, as well as their evaluated suitability for animal-appropriate housing of the declared target animal species, determined based on cage dimensions (floor area and height). Basis for the evaluation: online retail = 208 cage models, pet shops = 36 cage models, home improvement and garden centers = 13 cage models. ^1^Relative to housing maximal one golden hamster. ^2^Multiple listings possible.

Table 4 lists the product features of all assessed cage models and a separate as well as combined assessment of the floor area and height relative to housing one golden hamster (smallest of all required minimum cage dimensions for housing small mammal species) for the models purchasable in the German retail. The data show that between 100% (home improvement and garden centers) and 63.9% (pet shops) of the offered cage models were not even suitable for housing one golden hamster. When our evaluation included the declaration (i.e., the animal species for which the cage can be used) provided by the manufacturer, we found that many of the assessed cage models were declared not specifically for a target species but rather generally for “rodents and small animals.” With 50.5% (*n* = 105), the highest percentage of these nonspecifically declared cage models were sold via online retail. Considering the specific declaration for an animal species, we found that between 50.0% and 100% of the cage models, based on their dimensions, were not suitable for animal-appropriate housing of the declared animal species. Especially cage models declared for “more exotic” pets such as chinchillas and ferrets were not suitable for animal-welfare-compliant housing of two animals (Fig 1).

**Table 4:** Cage models purchasable from German retailers (online via internet shops, on site in pet shops or home improvement and garden centers) and their suitability for animal-appropriate housing of one golden hamster (minimal dimension requirement for housing a small mammal species) and specific design features.

			Online retail		Pet shop		Home improvement and garden center	
Assessed criterion			Number (*n*) and percentage distribution (%) of the cage models					
			*n*	%	*n*	%	*n*	%
Cage dimensions	FA^1^ ≥ 0.5 m^2^	Met	85	40.9	21	58.3	2	15.4
		Not met	123	59.1	15	41.7	11	84.6
	Height ≥ 0.5 m	Met	107	51.4	19	52.8	5	38.5
		Not met	101	48.6	17	47.2	8	61.5
	FA^1^ ≥ 0.5 m^2^, Height ≥ 0.5 m	Met	63	30.3	13	36.1	0	0.0
		Not met	145	69.7	23	63.9	13	100.0
Material	Cage top	Wood	0	0.0	0	0.0	0	0.0
		Metal	194	93.3	32	88.9	13	100.0
		Plastic	14	6.7	4	11.1	0	0.0
		Glass	0	0.0	0	0.0	0	0.0
	Cage bottom	Wood	30	14.4	0	0.0	3	23.1
		Metal	20	9.6	2	5.6	0	0.0
		Plastic	150	72.1	33	91.7	7	53.8
		Glass	8	3.8	1	2.8	3	23.1
**All assessed cage models**			**208**	**100.0**	**36**	**100.0**	**13**	**100.0**
Animal-welfare-adverse models^2^			183	88.0	29	80.6	13	100.0
Animal-welfare-compliant models^3^			25	12.0	10	27.8	0	0.0

^1^FA = Floor area

^2^do not meet one or more of the criteria listed in Table 1

^3^meet all of the criteria listed in Table 1.

Considering the used material, we noticed that most of the cage models had a base made of plastic (online retail: 72.1% [*n* = 150]; pet shops: 91.7% [*n* = 33]; home improvement and garden centers: 53.8% [*n* = 7]) and a wire top made of metal (online retail: 93.3% [*n* = 194]; pet shops: 88.9% [*n* = 32]; home improvement and garden centers 100.0% [*n* = 13]). However, we also found cage bases made of glass (online retail: 3.8% [*n* = 8]; pet shops: 2.8% [*n* = 1]; home improvement and garden centers: 23.1% [*n* = 3]) and cage tops made of plastic (online retail: 6.7% [*n* = 14]; pet shops: 11.1% [*n* = 4]).

In total, 183 (88.0%) of the 208 pet cage models available online did not meet at least one of the criteria listed in Table 1. Of these cage models, 29 were also offered in pet shops (80.6% of the sold models) and 13 in home improvement and garden centers (100% of the sold models).

### Evaluation of the assessed pet cages under animal welfare aspects

According to the “Fact Sheet on Animal-Welfare-Adverse Accessories in Pet Husbandry” published by the TVT, cages with an edge length of less than 50 cm + 100 cm (L + W) are not suitable for the permanent housing of small pets [30]. Undersized cages can reduce the wellbeing [15, 16, 48] and compromise the health of the animals [49, 50]. Several authors have pointed out that most of the commercially available pet husbandry systems are too small for animal-appropriate husbandry, albeit without referring to precise figures or empirical assessments [31, 51]. The results of our study show that of the 208 assessed cage models, 59.1% (*n* = 123) have a floor area of less than 0.5 m^2^ and thus should be considered animal-welfare-adverse according to the guidelines of the TVT [30].

Besides the cage floor area, the cage height also plays an important role in evaluating the cage size. A study on laboratory rats showed that the rats, if they have the possibility to stand upright, frequently and continuously show this behavior [52]. Thus, standing upright is part of their normal behavior, and the animal keeper or caretaker must enable them to act out this behavior, because according to § 2 of the German Animal Welfare Act [8], an animal keeper or caretaker must house the animal according to its behavioral needs. According to the “Expert Report on Minimum Requirements for Mammal Husbandry” (expert report on mammals) [32] and the guidelines of the TVT [41–47] and the BNA [33–40] for various small pet species, the minimally required cage height is 0.5 m. This requirement is not met by 48.6% (*n* = 101) of the herein evaluated 208 cage models, which thus are not suitable for small mammal husbandry.

**Fig 2:**
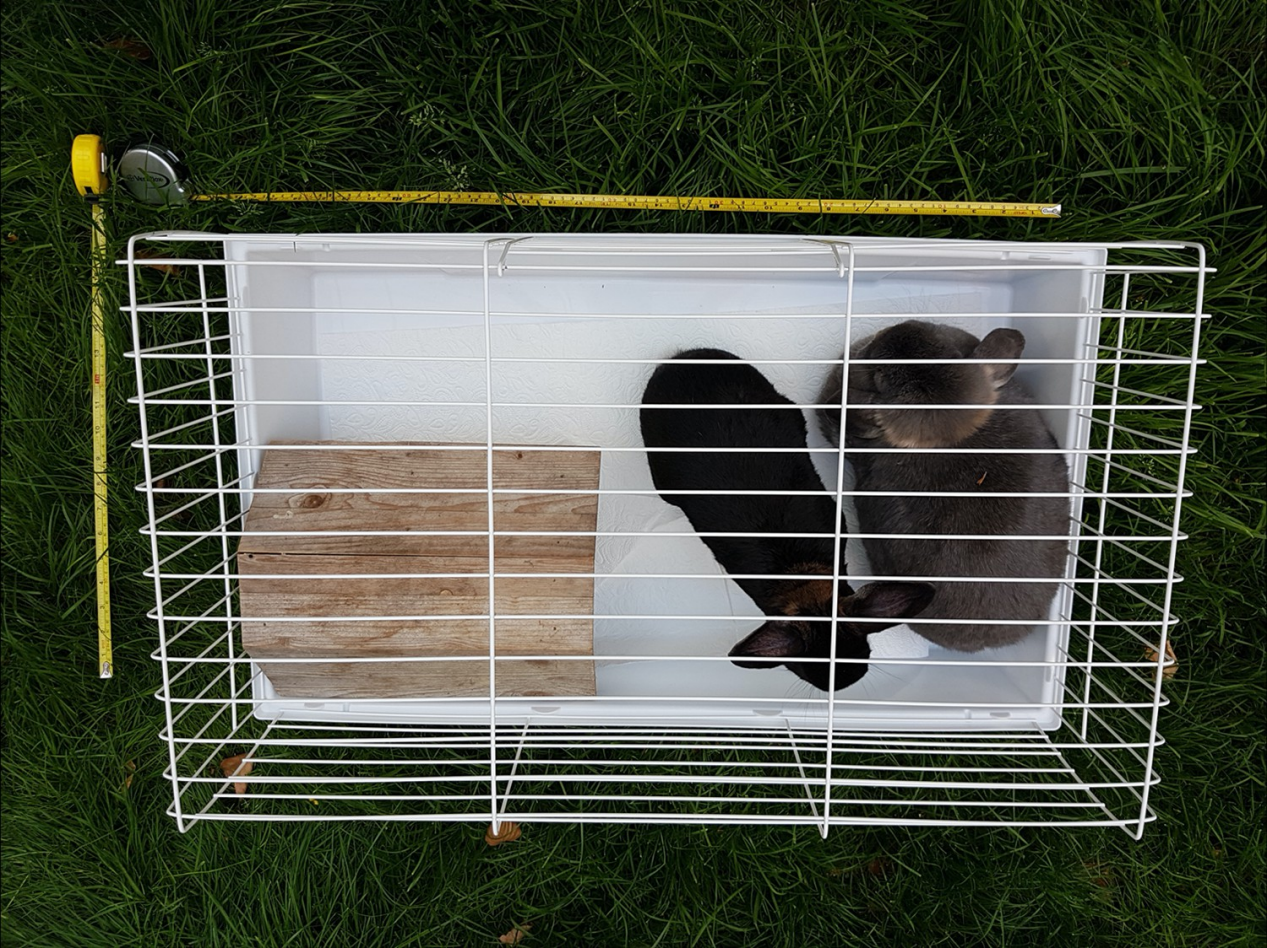
Pet cage available online, declared as suitable for rabbits, hares, and guinea pigs. Because every cage must offer a retreat possibility for the animals, a “hiding place” was put in the cage to demonstrate proportions. The cage dimensions provided by the manufacturer are: 80 cm × 46 cm × 36 cm (L × W × H). The black and the gray rabbit have a body weight of approx. 1.7 kg and 3.1 kg, respectively. (Photo: M. Ebner, 2018.).

Considering the cage floor area and height combined, we found that 69.7% (*n* = 145) of the assessed cage models are too small for housing a small mammal species. However, of these cage models, 43.4% (*n* = 63) were declared generally for “rodents and small animals.” This result highlights another problem: Only a few of the cage models available in (online) retail are unambiguously declared for one or several animal species and, if declared, are suitable for the declared animal species (Fig 2). For example, all the online-available cage models assessed for chinchillas (*n* = 21) and ferrets (*n* = 19) are too small for these animal species. It is furthermore remarkable that none of the cage models sold in home improvement and garden centers is suitable for housing small mammals. If manufacturers were required to declare the cages based on the cage dimension guidelines in the expert report on mammals [32], in the animal keeper fact sheets of the TVT [41–47], and in the animal species profiles of the BNA [33–40], animal keepers would have a much better chance to find a suitable cage for the animals they keep. Of course, the most recent version of these information sources must be considered, and in case of major differences, the expert report, fact sheet, or profile with the most recent date should be considered. For example, at the time of our data collection and evaluation, the recommendation of the TVT for housing two rabbits was a floor area of 150 cm × 60 cm (= 0.90 m^2^) and that of the BNA was 140 cm × 60 cm (= 0.84 m^2^). However, since 2019, the TVT has postulated a much larger floor area, namely 6 m^2^, for two rabbits [53]. Moreover, the cage declaration especially for rabbits must consider differences between the various breeds because a dwarf rabbit has different housing requirements than a German Giant does [54]. Cages that are too small for housing small mammals should no longer be offered in the pet supply market.

Besides the cage dimensions, the design and the used material also play an important role in the suitability of a cage for small mammal pet husbandry. Almost completely closed containers with only a few ventilation holes considerably limit the air circulation in the cage [30]. This is especially true for aquariums, which, for this reason, many authors consider unsuitable for housing rodents, rabbits, and ferrets [54–56]. Poor air exchange can lead to increased dust development and to heat and moisture buildup in the cage [50]. Because many small pet species, such as guinea pigs, rabbits, hamsters, and chinchillas, are particularly sensitive to heat [57–61], these animals can suffer a life-threatening heat stroke due to heat buildup [50, 60]. Another consequence of poor ventilation is the accumulation of harmful gases in the cage, which accumulate near the cage floor and thus around the animals [5]. Especially an increased ammonia concentration, originating from enzymatic degradation of urea in the excretions of the animals [62], can have negative effects on the animals’ health and, inter alia, lead to respiratory diseases [50, 60, 61]. In the present study, 6.7% (*n* = 14) of the 208 assessed cage models had a plastic top with only small ventilation slits, and another 3.8% (*n* = 8) were made of glass and had a grid cover. Although the TVT restrictively emphasizes that animal species originating from arid regions and producing little urine (such as gerbils) may be housed in aquariums [30], aquariums should no longer be offered in (online) retail for small mammals. Plastic cages that are closed on all sides and have inadequate ventilation slits should no longer be produced by manufacturers of pet supplies.

### Hay racks

The evaluation of hay racks was based on 72 models from 19 manufacturers. All of them could be purchased online. The assortment in the pet shops included 27 products from eight manufacturers and that in the home improvement and garden centers included 10 hay rack models from two manufacturers (Table 3).

The product features assessed on hay racks are listed in Table 5. Of the 72 assessed hay rack models, 48.6% (*n* = 35) were freestanding models, whereas 51.4% (*n* = 37) were designed for attachment inside or outside of the cage. A cover that would prevent the animals from lying in the hay rack was missing in 52.8% (*n* = 38) of the products. In 25.0% of the products, features such as horizontal bars, instable or foldable parts, or protruding metal spikes (etc.) bore a potential injury risk to the animals. In total, 40 (55.6%) of the 72 assessed hay rack models did not meet all the evaluation criteria listed in Table 1 (Table 5).

**Table 5:** Types and design features of feed or hay rack models purchasable from German pet supply retailers (online via internet shops, on site in pet shops or home improvement and garden centers).

		Online retail		Pet shop		Home improvement and garden center	
Assessed criterion		Number (*n*) and percentage distribution (%) of the feed or hay rack models					
		*n*	%	*n*	%	*n*	%
Type	Hay rack	64	88.9	24	88.9	7	70.0
	Feed ball	8	11.1	3	11.1	3	30.0
Attachment design	Freestanding	35	48.6	13	48.1	2	20.0
	Attachable	37	51.4	14	51.9	8	80.0
Attachment location if attachable	Outside of the cage	5	13.5	2	14.3	0	0.0
	Inside of the cage	32	86.5	12	85.7	8	100.0
Cover^1^	Present	33	51.6	14	58.3	2	28.6
	Not present	31	48.4	10	41.7	5	71.4
Material	Wood	39	54.2	16	59.3	2	20.0
	Metal	24	33.3	7	25.9	7	70.0
	Plastic	6	8.3	2	7.4	0	0.0
	Plastic and metal	3	4.2	2	7.4	1	10.0
Injury risk^2^	Yes	18	25.0	4	14.8	2	20.0
	No	54	75.0	23	85.2	8	80.0
**All assessed feed or hay rack models**		**72**	**100.0**	**27**	**100.0**	**10**	**100.0**
Animal-welfare-adverse models^3^		40	55.6	13	48.1	7	70.0
Animal-welfare-compliant models^4^		32	44.4	14	51.9	3	30.0

^1^Evaluated for hay racks only (online retail: *n* = 64, pet shops: *n* = 24, home improvement and garden centers: *n* = 7), not for feed balls.

^2^Injury risk due to, inter alia, poor manufacture and faulty design

^3^do not meet one or more of the criteria listed in Table 1

^4^meet all of the criteria listed in Table 1.

### Evaluation of the assessed hay racks under animal welfare aspects

Both herbivorous rodents and rabbits need daily ad libitum access to hay to assure dental abrasion, bowel movement, and formation of cecotropes [63]. The hay should be offered in a hay rack [64] because placement on the floor can lead to contamination of the hay with feces [65]. The hay racks must be designed in a way that they do not pose an obvious injury risk. Therefore, hay racks that are inside of the cage must be covered, so that the animals cannot jump inside and get injured [50, 64]. In the present study, a cover was missing in 51.6% (*n* = 33) of the assessed hay rack models, which thus must be considered animal-welfare-adverse [30, 31] and should no longer be sold. Additional injury risk originates from horizontal bars, transverse bars, or a large distance between vertical bars, posing the risk that the animals get stuck with their head or limbs and cannot free themselves. Especially feed balls, which accounted for 11.1% (*n* = 8) of the assessed hay rack models, should therefore generally be rejected due to animal welfare adversity and should be removed from the assortment of the manufacturers.

Besides injury risk, hay racks that are attached too high above the floor pose a problem because the animals have to eat with a raised head, which should be prevented by all means [64]. Prebble et. al., who investigated the influence of four types of diet for rabbits on the behavior of the animals, found that the rabbits preferred to eat hay in a natural grazing posture [66]. Accordingly, hay racks should be attachable near the floor, so that the animals are not restricted in their species-specific feed uptake. Moreover, all animals in a housing unit should be able to feed simultaneously [64]. Accordingly, the hay rack should be wide enough for all animals, or several hay racks must be provided. Furthermore, large hay racks should not be used for feed storage because microbial degradation processes in hay already begin after 24 hours [67], and thus hay should be replaced daily. Corresponding information for animal keepers could be printed on the product packaging or in a user manual. In addition to information about placement and maximal number of animals per hay rack, an indication of the appropriate animal species would help animal keepers sift through the extensive choice of products (72 models, excluding size and color variants) and find an animal-appropriate hay rack model for the animal species they keep. This accurate and complete information is especially important because we found 55.6% (*n* = 40) of the assessed hay rack models to be unsuitable for pet husbandry due to their design and the injury risk they pose to the animals.

### Running wheels

In total, 101 running wheel models from 16 manufacturers could be evaluated. All models could be purchased online. Furthermore, we found 36 running wheel models from 11 manufacturers in the visited pet shops and nine products from three manufacturers in the home improvement and garden centers (Table 3).

**Fig 3:**
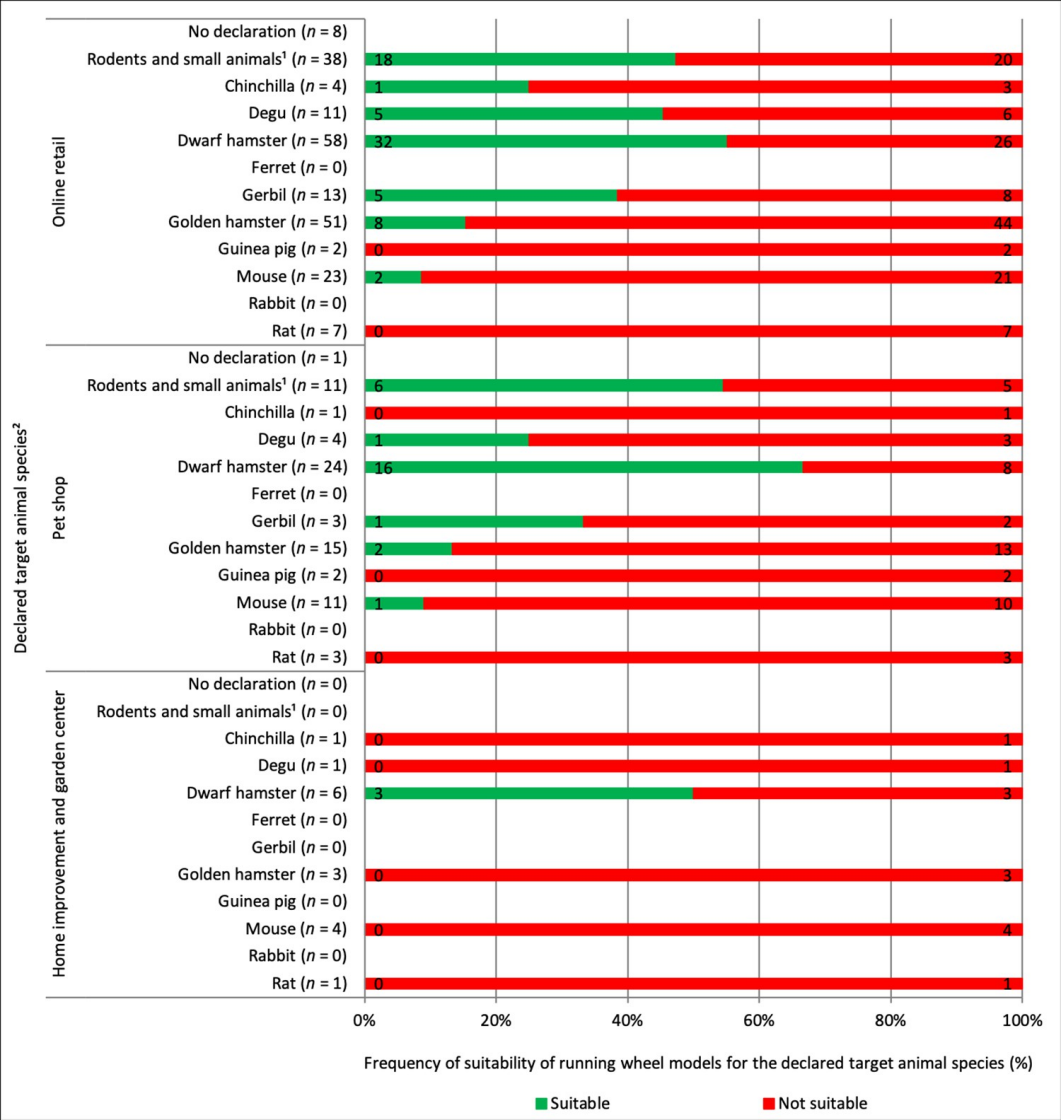
Running wheel models purchasable in German retail (online via internet shops, on site in pet shops or home improvement and garden centers) and declared for a target animal species, as well as their evaluated suitability for the declared target animal species, determined based on running wheel diameter. Basis for the evaluation: online retail = 101 running wheel models, pet shops = 36 running wheel models, home improvement and garden centers = 9 running wheel models. ^1^Checked for suitability for dwarf hamsters. ^2^Multiple listings possible.

The product features of the assessed models are listed in Table 6. Of all purchasable running wheel models, 42.6% (*n* = 43) had a diameter smaller than 20 cm and thus were not suitable for any of the small mammal species, and another 50 models (49.5%) according to their size (≥20 cm and <30 cm in diameter) should be used exclusively as an accessory for dwarf hamsters [30]. Conversely, this result implies that only 7.9% of the offered models were suitable for husbandry of other small mammal species. Including the declared target animal species in the evaluation, we found the majority (66.3%; *n* = 67) of the assessed running wheel models to be declared for several animal species, but we also found products without declaration of the target animal species (7.9%; *n* = 8). According to the product details provided by the manufacturers, 57.4% (*n* = 58) of the 101 assessed running wheel models should be suitable enrichment material for dwarf hamsters. However, according to our analysis, this was the case for only about one half (55.2%; *n* = 32) of these 58 models. Of the 51 running wheel models declared for golden hamsters, only 15.7% (*n* = 8) had a diameter large enough (≥30 cm) for this animal species (Fig 3).

**Table 6:** Design features of running wheel models purchasable from German pet supply retailers (online via internet shops, on site in pet shops or home improvement and garden centers).

		Online retail		Pet shop		Home improvement and garden center	
Assessed criterion		Number (*n*) and percentage distribution (%) of the running wheel models					
		*n*	%	*n*	%	*n*	%
Diameter	<20 cm	43	42.6	13	36.1	4	44.4
	≥20 cm und <30 cm	50	49.5	21	58.3	5	55.6
	≥30 cm	8	7.9	2	5.6	0	0.0
Material	Wood	36	35.6	13	36.1	5	55.6
	Metal	29	28.7	7	19.4	0	0.0
	Plastic	36	35.6	16	44.4	4	44.4
Injury risk	Spaced-apart rungs as running surface	15	14.9	3	8.3	0	0.0
	Axle side open^1^	28	27.7	8	22.2	0	0.0
**All assessed running wheel models**		**101**	**100.0**	**36**	**100.0**	**9**	**100.0**
Animal-welfare-adverse models^2^		87	86.1	31	86.1	9	100.0
Animal-welfare-compliant models^3^		14	13.9	5	13.9	0	0.0

^1^The side with the wheel suspension; injury risk due to wheel suspension or transverse braces

^2^do not meet one or more of the criteria listed in Table 1

^3^meet all of the criteria listed in Table 1.

We found a possible injury risk on 14.9% (*n* = 15) of the running wheel models due to spaced-apart rungs serving as running surface and on 27.7% (*n* = 28) due to the axle side not being closed. In the models with open axle side, either the backside (running wheel attached on only one side) or both sides (running wheel held by a stand) were open. Considering all evaluation criteria listed in Table 1, we found that of the 101 evaluated running wheel models, 87 (86.1%) did not meet at least one criterion (Table 6).

### Evaluation of the assessed running wheels under animal welfare aspects

Only few empirical studies have dealt with the influence of a running wheel on the wellbeing and health of small mammals kept as pets. Gebhardt-Henrich et al. examined how the use of a running wheel affects female golden hamsters kept under pet husbandry conditions; they concluded from the results that a large and injury-risk-free running wheel can be recommended for golden hamster husbandry [68]. Other authors also recommend animal-appropriate running wheels as enrichment objects for golden and dwarf hamsters, mice, gerbils, chinchillas, and degus [50, 55, 64, 69, 70].

To assure that the running surface does not pose an injury risk, it should be completely solid. On non-solid running surfaces such as spaced-apart rungs the animals’ limbs could get caught between the rungs and incur fractures [30, 61]. In addition, as also found for running surfaces made of metal rods and covered with a plastic mesh, injuries on the paws are possible [28]. Regarding the assembly of the running wheels, it is important that they stand securely and that the wheel turns easily and without side noise [50]. Running wheels that are attached on only one side must have a closed backside to prevent the animals from getting caught between cylinder and wheel suspension and getting injured [30]. A running wheel with stabilizing transverse braces in combination with an open axle side bears the risk that the animal gets its limbs or head caught. Severe injuries, even severed limbs, can be the consequence [5]. In summary, an animal-welfare-compliant running wheel should be designed without transverse braces, with a solid running surface, and with a closed axle side [50, 51, 60]. If these conditions are not met, the running wheel bears an increased injury risk and must be considered animal-welfare-adverse [30, 31]. As long ago as 2000, Steinigeweg reported that many commercially available running wheel models bear a high injury risk due to their design features [5]. In the present study, 14.9% (*n* = 15) of the assessed 101 running wheel models had spaced-apart rungs as a running surface and 27.7% (*n* = 28) either had an open backside or had both sides open with the wheel being stabilized by transverse braces.

In addition to their design, the diameter of running wheels is an important aspect in evaluating their animal welfare compliance. An undersized running wheel can lead to abnormal spinal curvature, spinal disc damages, and development of spondylarthritis [60]. Running wheels with a diameter smaller than 20 cm (for dwarf hamsters) or 30 cm (for all other small mammals) are considered animal-welfare-adverse by the TVT [30]. Of the 101 running wheel models assessed in this study, 42.6% (*n* = 43) had a diameter smaller than 20 cm and thus are not suitable as enrichment object for any small mammal. However, 20 (46.5%) of these were declared generally for “rodents and small animals.” As also found for pet cages, many running wheel models are not properly declared. When they are declared for an animal species, their diameter is often unsuitable for the declared animal species. Of the 51 running wheel models that were declared for golden hamsters, only 15.7% (*n* = 8) had a diameter of 30 cm or more and are thus animal-welfare-compliant according to the TVT guidelines [30].

**Fig 4:**
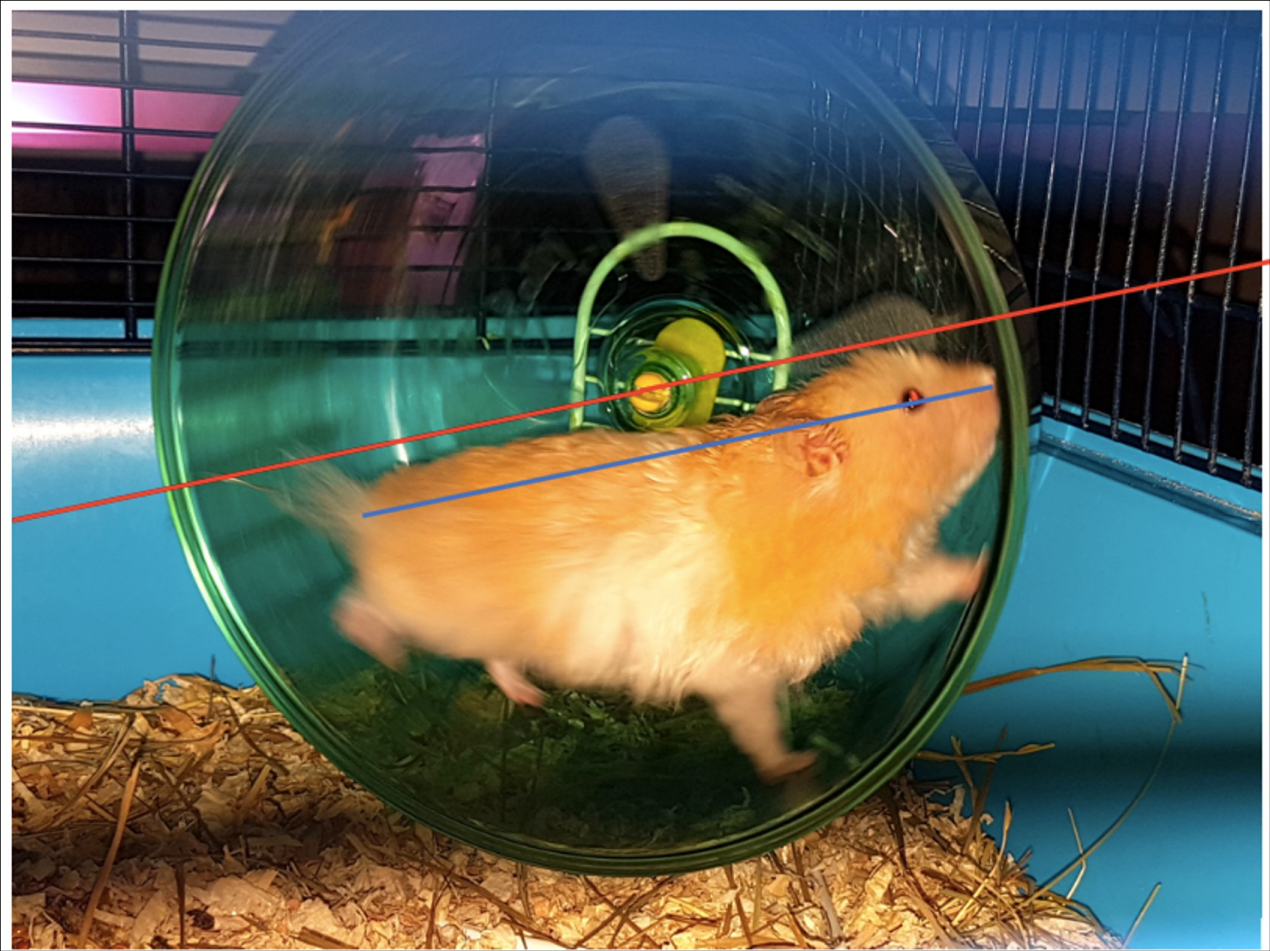
A running wheel with markedly undersized diameter. Red line: Desired diameter (= 2 × body length) of the running wheel. Blue line: Approximate body length of the pictured animal. (Photo: M. Ebner, 2018.).

However, some of the running wheel dimensions recommended by the TVT must be critically questioned. To prevent strain on the spine, the animal should be able to run with a straight back in the running wheel [71]. To allow a straight back, the running wheel diameter must be twice as long as the body length [72]. Because golden hamsters can have a body length of 15–18 cm [38], large animals would require a running wheel diameter of at least 36 cm (Fig 4). A running wheel suitable for fancy rats, which have a body length of approx. 40–46 cm [40], should thus have a diameter of almost 1 m [73]. Because rats furthermore have a convexly curved spine and a very long tail, the common commercially available running wheels are unsuitable for rats and should not be used in pet husbandry [64, 74].

In total, 86.1% (*n* = 87) of the 101 assessed running wheel models had an undersized diameter for the declared animal species or bore an injury risk and thus are unsuitable enrichment object for small mammal pets. For animal welfare reasons, running wheels whose design poses a substantial injury risk to the animals should no longer be sold in (online) retail. Moreover, to help animal keepers decide whether a running wheel is suitable for the kept animal species, a declaration duty should be introduced for running wheels. In determining the appropriate animal species, manufacturers should have to comply with the following rule of thumb: diameter = 2 × body length. If the relevant literature indicates that running wheels should not be offered for a certain animal species, a corresponding warning (e.g., not suitable for fancy rats) should be printed on these running wheels.

### Exercise balls

The product group exercise balls included 25 articles from 16 manufacturers, and all of them could be purchased online. Pet shops sold three exercise ball models from two manufacturers. Home improvement and garden centers did not offer exercise balls in their assortment (Table 3).

Of the 25 assessed products, all were made of plastic and about half of them (48.0%; *n* = 12) were transparent or transparently colored. The other articles were available in various colors. The average diameter of the offered exercise ball models was 17.3 cm. The smallest diameter was 12 cm (seven models) and the largest 32 cm (one model). Exercise balls generally poses a high risk of injury due to the possibility that the animals are bumping against a wall or object or falling off of an elevated surface, which is why none of the examined balls met the evaluation criterion listed in Table 1.

### Evaluation of the assessed exercise balls under animal welfare aspects

Many authors have emphasized that hamster balls or exercise balls or similarly designed toys, such as hamster cars, should not be used in pet husbandry due to animal welfare concerns [5, 30, 31, 50, 70, 75, 76]. Retreat and orientation possibilities are not given, causing considerable stress for the animals [30, 31], and the species-specific locomotion of the animals is considerably restricted, which is forbidden according to § 2 of the German Animal Welfare Act [8]. Moreover, the exercise balls are usually made of plastic and have only small ventilation holes, leading to poor ventilation [30, 31] and consequently to a lack of oxygen in the ball [31, 50]. Furthermore, the ball bumping against a wall or object or falling off of an elevated surface bears a considerable injury risk for the animals [5, 30, 31, 50]. For these mentioned reasons, exercise balls and hamster balls should be removed permanently from the assortment in retail. The check list “Dangerous Accessories for Pets” (Gefährliches Zubehör für Heimtiere), compiled jointly by the ZZF and the IVH, also lists hamster balls and hamster cars as animal-welfare-adverse accessories that should not be commercially available [77].

### Harnesses and leashes

In the product group harnesses and leashes, the assortment of the online retailers included 50 models from 17 manufacturers (Table 3). Not all visited pet shops (*n* = 50) or home improvement and garden centers (*n* = 13) offered harnesses and leashes. We found in 21 pet shops 13 models from three manufacturers and in four home improvement and garden centers four products from one manufacturer.

Of the offered harnesses, eight models were declared generally for “rodents and small animals.” If the articles were declared specifically for one or several animals species, rabbits were listed most often (*n* = 25), followed by guinea pigs (*n* = 9), ferrets (*n* = 6), rats (*n* = 4), and hamsters (*n* = 2). Because of the general risk of injury they pose due to reflex-like flight attempts of the animals on the leash or constrictions in the chest or belly area due to improperly fitting or incorrectly applied harnesses, no harnesses and leashes evaluated in the study met the evaluation criterion listed in Table 1.

### Evaluation of the assessed harnesses and leashes under animal welfare aspects

According to the DTschB, the wearing of harnesses and leashes should generally be rejected for small rodents and rabbits due to the associated stress [31]. The TVT also describes harnesses and leashes as absolutely inappropriate for most rodents because of the possible injury risk, among other factors; mentioned exceptions include, for example, tame rabbits that are slowly habituated to wearing a harness [30]. Most small pets (except ferrets) are prey animals that can show a startle response to certain situations and react with flight or escape attempts [50, 51, 64, 65]. If, in these cases, the animals are leashed, they may incur injuries due to reflex-like flight attempts. Especially rabbits have a very delicate spine, and spine fractures or traumata occur frequently in practice [70]. Furthermore, many small pets show a marked thigmotaxis, i.e., as part of their natural behavior they avoid open areas und stay near structures (walls, bushes, etc.) [51, 64]. The use of harnesses and leashes can considerably restrict the animals in their natural locomotion behavior, and such restriction is forbidden according to § 2 of the German Animal Welfare Act [8] and thus should be prevented by all means. Moreover, if a harness does not fit properly or is applied improperly, the abdominal strap can cause constrictions in the chest or belly area and consequently lead to respiratory restrictions. Especially in rabbits and guinea pigs, the rib cage should never be tightly girdled because the last three, free-ending ribs could cause a lung compression [50, 64]. Thus, due to the injury risk they pose, harnesses and leashes for small rodents and rabbits are to be considered animal-welfare-adverse and should not be used. Exceptions for tame rabbits, as described by the TVT [30], can only be approved in an absolutely controllable environment. However, the question arises whether the product diversity (50 models excluding color variants) found in this study encourages pet keepers to buy harnesses and leashes for animals they keep, and, thus, whether these products in general should no longer be sold in retail.

### Tube systems

In total, we assessed 14 tube system models from 13 manufacturers. All tube system models could be purchased online. Three models from two manufacturers were available in pet shops and none was sold in home improvement and garden centers (Table 3).

For 3 of the 14 tube systems, dimensions were not given. For the remaining 11 assessed products, the tube diameter was on average 6.5 cm (SD: 2.0 cm; minimum: 5.1 cm, maximum: 11.0 cm). The most frequently used material for tube systems was plastic, with 92.9% (*n* = 13). Only one system was made of cellulose. Half of the manufacturers (*n* = 7) provided information on the presence of ventilation holes, but if ventilation holes were present (*n* = 6), information on their number and size was missing. Four manufacturers specified that the tubes had a profile, whereas the remaining manufacturers did not provide information about the inner tube surface. Due to the injury risk, the risk of the animals getting stuck in undersized tubes and thus being restricted in their natural movement behavior, and the insufficient ventilation possibilities in the systems, no tube system did met at least one of the criteria in Table 1.

### Evaluation of the assessed tube systems under animal welfare aspects

Tube systems, due to the injury risk they pose und the poor ventilation, are considered animal-welfare-adverse by the DTschB and the TVT [30, 31]. Other authors also emphasize that tube systems made of plastic are absolutely inappropriate for animal husbandry [50, 70, 76]. Of the 14 systems assessed in our study, 13 consisted of plastic, and only six manufacturers had included in the product description that the tubes have ventilation holes. However, even when ventilation holes are present, a sufficient air exchange in the tube system is not guaranteed. The poor ventilation can lead to heat and moisture buildup [50]. Furthermore, because the tubes are difficult to clean, pathogens can propagate in them [31] and enzymatic degradation of urea can lead to increased accumulation of ammonia [62]. Both factors pose health hazards to the animals.

Another animal-welfare-relevant problem of the tube systems is the injury risk they bear. Hamsters have a very limited height perception [69, 72]. Thus, they can easily fall off of elevated surfaces and incur severe injuries [64]. In tube systems, the risk of falling is considerably increased by two factors. On the one hand, unnatural slopes can be built during assembly [5, 30, 31], on the other hand, some tube systems have no profile and thus a very smooth (slippery) inner surface. In the present study, we found that the products from only 4 of 14 manufactures included the information that the tubes had a profile, which gives the animals better foothold. Furthermore, the tube diameter can pose a problem. The tubes must be large enough so that the animals can freely move in them and even turn around. The Swiss animal welfare organization “Zürcher Tierschutz” postulates for golden and dwarf hamsters “hiding places” with large enough entrances and specifies the required minimal diameter of the opening to be 8 cm for golden hamsters and 6 cm for dwarf hamsters [72]. Applying these values as minimal tube diameter for the commercially available tube systems, eight of the herein evaluated systems (no size information, diameter less than 6 cm) are unsuitable for both golden hamsters and dwarf hamsters.

Due to the injury risk, the risk of the animals getting stuck in undersized tubes and thus being restricted in their natural locomotion behavior, and the insufficient ventilation options in the systems, tube systems must be considered animal-welfare-adverse and should no longer be available in retail. Individual tubes that are well ventilated and large enough can be used as retreat possibilities or as connections between two cages [30, 64]. However, these tubes should have a maximum length of twice the body length of the animal and should be made of natural materials [31].

### Hamster bedding

Table 3 shows the number of hamster bedding products offered online and on site. In total, we found 10 products from 8 manufacturers.

Considering the used materials, the hamster bedding products can be categorized in four groups. Four products contained cotton or cotton fibers, three consisted of natural fibers or were listed as natural products, two products consisted of environmentally friendly fibers, and one product, according to the manufacturer, was made of a special germ-free material. The information “completely digestible” was found on almost all products. However, the color variants from one manufacturer differed in this regard. Brown hamster bedding from this manufacturer was specified as completely digestible, whereas the white variant included no information on digestibility; upon inquiry, it was specified as non-digestible. Considering all evaluation criteria listed in Table 1, seven of the evaluated hamster bedding met the criterion “digestibility” and did not contain synthetic material or synthetic fibers.

### Evaluation of the assessed hamster bedding under animal welfare aspects

Hamsters, as well as other rodents, should be provided with material for nest building [41, 45, 75, 76]. For this purpose, special hamster bedding is offered commercially. If it consists of synthetic fibers, it can (due to its tear strength) cause cheek pouch blockage, digestive problems, or strangulation of the limbs [5, 76]. Therefore, the TVT considers synthetic hamster bedding animal-welfare-adverse [30], whereas other authors consider hamster bedding without the indication “fully digestible” unsuitable for pet husbandry [5, 31]. However, Ewringmann and Glöckner point out that although hamster bedding indicated as “digestible” does not cause injuries in the digestive tract, it can cause strangulation of toes and limbs; therefore, they consider hamster bedding principally unsuitable for hamsters [60]. Rother and Lazar also describe bedding material declared as “digestible” as being unsuitable for hamster husbandry [76]. Because other materials, such as clean and high-quality hay and straw or unbleached cellulose can also serve as nesting material [60, 75], hamster bedding, due to its inherent injury risk, should principally not be used in pet husbandry and thus should be taken off the market by the manufacturers.

## Conclusions

Our study results show that a multitude of differently designed pet housing systems and accessories are offered in the German pet market. The diversity in the assortment has previously been pointed out by other authors [5, 7]. Due to the diverse choice of products, the herein presented results cannot represent a complete assessment of all offered products, despite our intensive research.

In Germany, no legal regulations exist regarding the design and declaration of the accessories. However, according to § 1 of the German Animal Welfare Act, it is forbidden “to inflict pain, suffering, or injuries without reasonable cause on an animal.” Furthermore, according to § 2 Section 2 of the German Animal Welfare Act, the animal keeper or caretaker is not allowed “to restrict the animal’s possibility for species-specific movement in a way that inflicts pain or avoidable suffering or injuries on the animal” [8]. We evaluated the accessories offered in German (online) retail under these aspects and consulted the “Fact Sheet on Animal-Welfare-Adverse Accessories in Pet Husbandry” published by the TVT [30] and the “Position Paper on Animal-Welfare-Adverse Accessories and Toys” published by the DTschB [31]. Our results show that the majority of the offered pet cage models and accessories is not suitable for pet husbandry and should be considered animal-welfare-adverse.

The majority of pet cage models and accessories offered in German retail are unsuitable for pet husbandry and must be considered animal-welfare-adverse. This conclusion applies to 55.6% (*n* = 40) of the 72 assessed hay rack models, 82.7% (*n* = 172) of the 208 assessed pet cage models, and 86.1% (*n* = 87) of the 101 assessed running wheel models. The articles offered in the products categories exercise balls, harnesses and leashes, tube systems, and hamster bedding must generally be rejected due to animal welfare concerns. Moreover, we found clear shortcomings in the article declaration. They make it difficult for pet keepers to distinguish between suitable and unsuitable accessories for the animals they keep. It is also problematic that, especially on the internet, one can find images of cages and accessories with animals in or on them. Such images suggest to the pet keeper that the shown objects allow for animal-appropriate husbandry. Several authors have previously pointed out that the way pets are held in pet shops influences pet keepers in the decision on how to house the pets at home [18, 19, 64].

To assure that less animal-welfare-adverse and furthermore properly declared accessories are offered in German retail, not only the retailers but first and foremost the manufacturers must be held accountable. A declaration duty for manufacturers could provide animal keepers with important product information and facilitate their decisions in finding suitable products for the animals they keep. Importantly, the product information on the packaging should clearly indicate the animal species for which the product can be used. Additional user information relevant to the product category must also be displayed on the packaging. Such information includes assembly directions, suitability for young or geriatric animals, maximum number of animals per article, etc.

A voluntary product certification issued by an independent, qualified third party, following the example set by the Austrian “animal welfare label” [12], would allow assessing animal welfare compliance of articles before they become commercially available. Such assessment needs well-defined and transparent test criteria based on current empirical findings. If a product is unsuitable for pet husbandry because it does not meet the set requirements, it must be considered animal-welfare-adverse and rejected. If the assessment shows that the article can only be approved under certain conditions, corresponding requirements must be imposed on the manufacturer, especially regarding the article declaration (e.g., not suitable for young animals). Animal-welfare-compliant articles could be indicated with a corresponding label, allowing pet keepers to easily recognize them. In Germany, such a certification system could be established, for example, through an authorized, independent governmental office. Alternatively, this task could be taken on by private institutions with regulatory authority.

Another option to especially reduce the demand for animal-welfare-adverse products is to improve the knowledge of pet keepers regarding species-specific and animal-appropriate housing of the animals they keep. For example, current and prospective pet keepers could be required to provide a mandatory proof of subject knowledge. Moreover, knowledge transfer could be facilitated via a certified internet platform that provides current scientific information on animal-welfare-compliant husbandry of individual animal species. Furthermore, a legal order or directive on pet husbandry could be established [17]. Finally, empirical studies examining the influence of various husbandry conditions on the wellbeing of pets should be supported to increase the knowledge on species-specific and animal-appropriate pet husbandry. Although studies on several related topics regarding laboratory and farm animal husbandry already exist, the findings are not always transferable to pet husbandry [22].

Many of the articles assessed in the study are available online and can be purchased worldwide. Articles found to be in violation of animal welfare in this study can endanger the welfare of animals and, in the worst case, lead to serious injury and death of the animal. Therefore, the recommendations for action proposed in this paper are transferable to other countries.

## Supporting information

S1 Data (XLSX)
